# Coarctation of the Aorta - The Current State of Surgical and Transcatheter Therapies

**DOI:** 10.2174/1573403X113099990032

**Published:** 2013-08

**Authors:** Jeffrey E Vergales, James J Gangemi, Karen S Rhueban, D Scott Lim

**Affiliations:** 1 Children’s Hospital Heart Center, Department of Pediatrics, University of Virginia,; 2 Children’s Hospital Heart Center, Department of Surgery, University of Virginia, Charlottesville, VA 22908

**Keywords:** Balloon angioplasty, coarctation of the aorta, stent, surgery.

## Abstract

Aortic coarctation represents a distinct anatomic obstruction as blood moves from the ascending to the descending aorta and can present in a range of ages from infancy to adulthood. While it is often an isolated and discrete narrowing, it can also be seen in the more extreme scenario of severe arch hypoplasia as seen in the hypoplastic left heart syndrome or in conjunction with numerous other congenital heart defects. Since the first description of an anatomic surgical repair over sixty years ago, an evolution of both surgical and transcatheter therapies has occurred allowing clinicians to manage and treat this disease with excellent results and low morbidity and mortality. This review focuses on the current state of both transcatheter and surgical therapies, paying special attention to recent data on long-term follow-up of both approaches. Further, current thoughts will be explored about future therapeutic options that attempt to improve upon historical long-term outcomes.

## INTRODUCTION

Coarctation of the aorta has previously been defined as a congenital stenosis of the aorta, often occurring in the juxtaductal position. It can be associated with other lesions including a patent ductus arteriosus [1], ventricular septal defect [2], bicuspid aortic valve [3] and a variety of left-sided obstructive lesions including hypoplastic left heart syndrome [4, 5]. The first pathologic description of aortic coarctation was documented by Morgagni in 1760 [6] with initial operative outcomes published in 1945 by Crafoord and Gross [7, 8]. Since then, numerous excellent reviews and primary literature have described the embryologic theories [9, 10], natural history [11] and clinical diagnosis of this disease [12, 13]. This review will briefly summarize the current anatomic and diagnostic issues before focusing on the various therapeutic options and current knowledge of long-term outcomes of coarctation of the aorta. 

## ANATOMIC CONSIDERATIONS

**Fig. (1):**
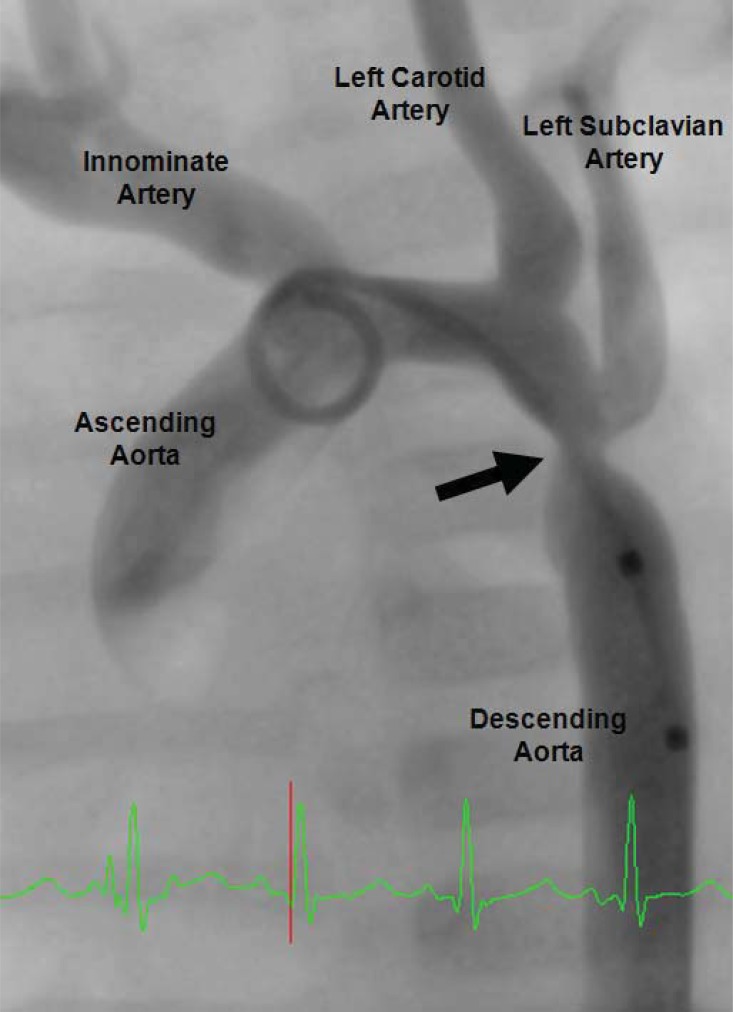
Angiogram of aortic coarctation in an infant. The solid black arrow denotes discrete coarctation just distal to the takeoff of the left subclavian artery in what is commonly the juxtaductal position.

Coarctation of the aorta is often a discrete obstruction commonly located in the descending thoracic aorta. More specifically, it is usually located in the juxtaductal position immediately distal to the left-subclavian artery in a left-sided arch (Fig. **1**). Infrequently, it can also exist in the transverse aortic arch and abdominal aorta, or be a part of a long-segment arch hypoplasia as seen in various left-sided obstructive lesions such as hypoplastic left heart syndrome [5]. When it is located in the more common juxtaductal position, it is frequently associated with distal displacement of the left subclavian artery which can easily be seen by echocardiography or angiography as an increased distance between the takeoff of the left carotid artery and that of the left subclavian artery. The right subclavian artery can often arise aberrantly distal to the origins of the other head and neck vessels [14]. While it can exist in isolation, its frequent association with other congenital defects such as a patent ductus arteriosus, ventricular septal defect, bicuspid aortic valve, and left-sided obstructive lesions makes it prudent to identify the complete intracardiac anatomy [1-5, 14].

## DIAGNOSIS

The hallmark clinical finding in coarctation of the aorta is hypertension proximal to the lesion, with diminished blood pressure distal to the obstruction. Consequently, clinical diagnosis can be made by the presence of diminished lower extremity pulses, differences in timing between upper extremity central pulses (often brachial) and lower extremity central pulses (often femoral), or the presence of a supine arm-leg blood pressure gradient. In patients with an aberrant right subclavian artery, all four extremities may be supplied by vessels distal to the obstruction, thus making diagnosis difficult. In neonates, a patent ductus arteriosus may help by limiting the degree of physiologic obstruction around a juxtaductal coarctation, but at the expense of possibly obscuring the diagnosis. Therefore, a neonatal coarctation may not manifest itself until the ductal tissue fully constricts [9].

**Fig. (2):**
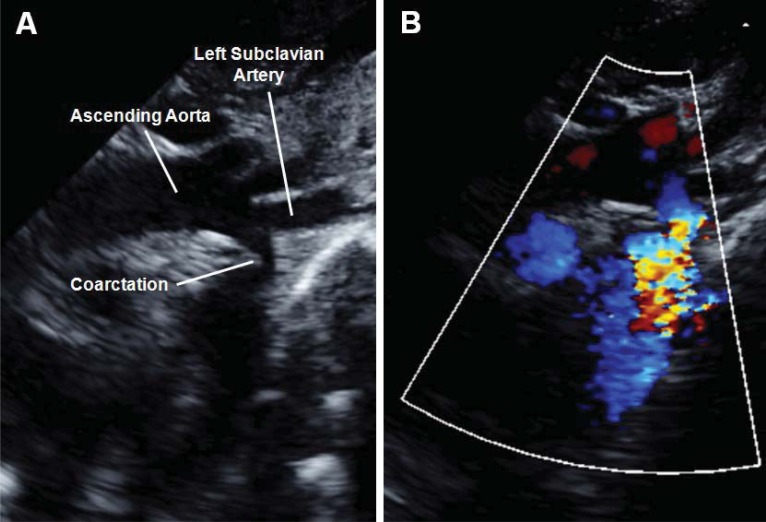
Panel A shows a two-dimensional transthoracic echocardiogram from the suprasternal view demonstrating a discrete narrowing just distal to the take-off of the left subclavian artery. This individual did not have a displaced left subclavian artery but instead had an aberrant right subclavian artery originating near the coarctation (not shown). Note the continuous high velocity color Doppler signal across the hypoplastic coarctation segment (Panel B).

**Fig. (3):**
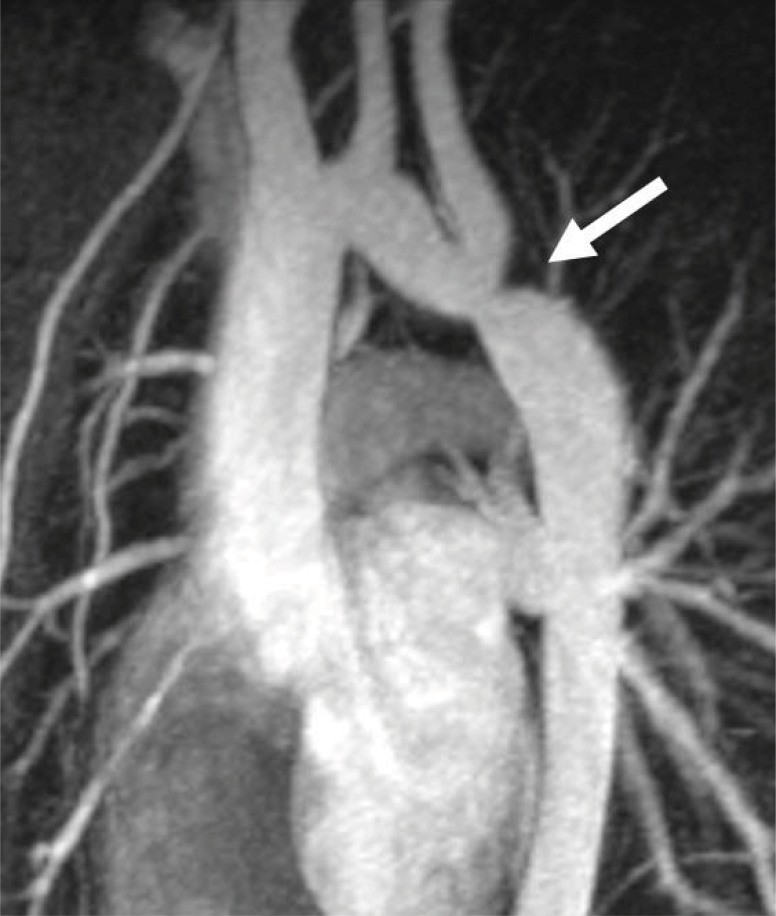
Magnetic resonance imaging of a 13 year-old female with a history of coarctation repair by end-to-end anastomosis as an infant. Hypertension, with a 20 mmHg extremity gradient seen during follow-up, led to further evaluation which revealed a discrete area of recoarctation (white arrow). She successfully underwent endovascular stenting and is currently doing well.

Transthoracic echocardiographic techniques have become the standard in confirming clinical suspicion of coarctation [12, 13]. This can best be demonstrated in the suprasternal view by demonstrating a narrowed aortic lumen in addition to measuring of the Doppler derived gradient across the coarcted segment (Fig. **2**). In older patients where acoustic windows may be suboptimal, it can be difficult to visualize the descending aortic segment of concern. In these patients, magnetic resonance imaging (Fig. **3**) and less often computed tomography scanning has become the modality of choice in the evaluation and management of both native and recurrent coarctations [15-19]. This includes being able to make accurate predictions about severity of the coarctation gradient as seen at the time of cardiac catheterization [20]. 

## THERAPEUTIC CONSIDERATIONS

The indications for therapeutic intervention for coarctation are clear for the neonate struggling with or at risk for hemodynamic collapse from significant obstruction or with evidence of multiple left-sided obstructive lesions. However, indications for intervention are less clear for asymptomatic patients with mild hypertension and a documented blood pressure gradient between their upper and lower extremities. Campbell’s natural history study on coarctation suggests an increase in mortality and median lifespan of just 31 years secondary to congestive heart failure, aortic rupture and an increased risk of endocarditis for those who remain unrepaired [11]. Most authors still utilize a blood pressure gradient greater than 20 mmHg as a significant coarctation warranting intervention. For recurrent chronic obstructions, however, the presence of significant aortic collaterals may reduce the perceived gradient in comparison to anatomic severity, making additional imaging modalities necessary in the initial diagnosis and follow-up of these patients [21]. 

## HISTORICAL THERAPEUTIC OPTIONS

### Resection with End-to-End Anastomosis

The first surgical therapy documented for coarctation was performed separately by Crafoord and Gross in 1945 through a lateral thoracotomy and involved resection of the coarcted segment followed by a direct suture anastomosis of the transected ends [7, 8]. High recoarctation rates complicated this approach, particularly when performed in neonates [22-25]. Consequently, it has for the most part been abandoned for newer surgical techniques that do not involve a circumferential suture line. 

### Patch Aortoplasty 

Concerns about high recoarctation rates following end-to-end anastomosis led to attempts to augment the coarcted segment with prosthetic material [26]. Similar to the initial approach, after ligation and division of ductal tissue through a thoracotomy, the aorta is cross-clamped proximal and distal to the coarcted segment. A longitudinal incision is then performed along the lateral aortic wall through the obstructed area and a prosthetic patch is sutured across the incision which enlarges the vessel lumen. Initially, the prosthetic material used was made of Dacron [27]. While this technique originally demonstrated a lower rate of recoarctation [28], it fell out of favor when significant aneurysms were seen developing on the wall opposite the prosthetic graft in 20-40% of cases [27, 29-31]. Polytetrafluoroethylene was thought to reduce the mechanical factors believed to lead to aneurysm formation by being more distensible than Dacron [32], but still demonstrated a recoarctation rate of 25% along with a 7% aneurysm rate [33].

Patch aortoplasty is still being used, however, in the setting of more complex aortic arch reconstructions. This is especially evident in severe arch hypoplasia, as seen in infants with hypoplastic left heart syndrome, where the transverse arch is augmented with cryopreserved pulmonary artery allograft [34]. Recoarctation rates upwards of 30% in these patients are currently being seen [35, 36], but long-term aneurysm formation at this time has not been described.

### Subclavian Flap Aortoplasty

This technique, which utilizes one’s own tissue, was first described by Waldhausen [37] in 1966 but did not move into mainstream practice until many years later in an attempt to reduce long-term complication rates. Like the other techniques, it is performed through a lateral thoracotomy with the aorta being cross-clamped proximal to the left subclavian artery and distal to the coarctation. After ligation and division of the ductus arteriosus, the left subclavian artery is ligated near the take-off of the left vertebral artery and an incision is made along its underside down onto the aortic isthmus and across the coarctation. This flap of tissue is then folded down on the now-incised coarctation segment and anastomosed to enlarge the vessel lumen. A variation of this technique for arch hypoplasia involves the subclavian flap folded in the reverse onto the proximal hypoplastic portion [38]. 

**Fig. (4):**
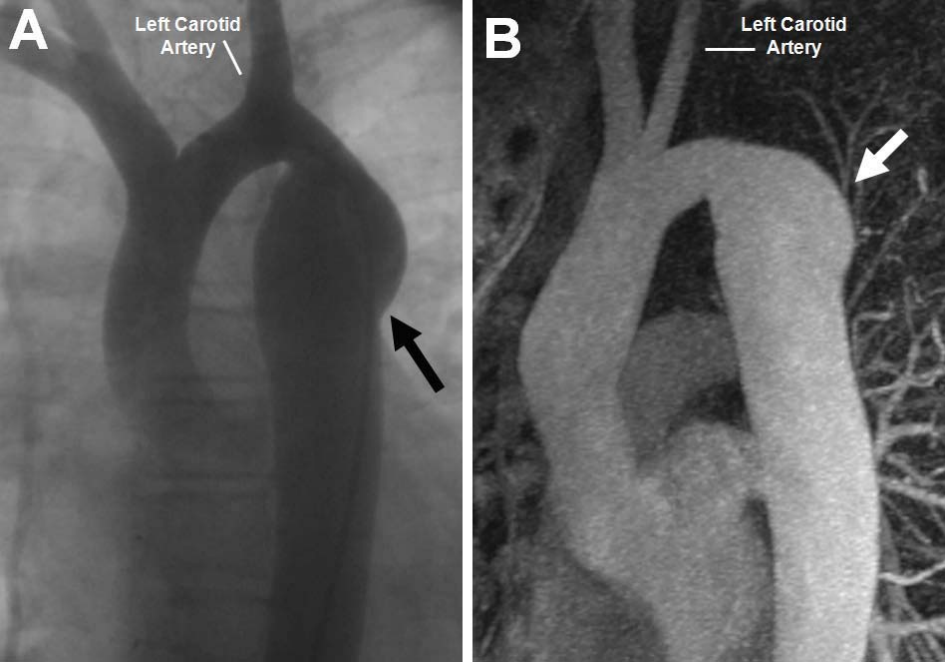
Angiogram demonstrating aneurysm development (Panel A, black arrow) in a 13 year-old patient who previously underwent subclavian flap aortoplasty for coarctation of the aorta. Magnetic resonance imaging of a 30 year-old patient in Panel B also demonstrates aneurysm formation (white arrow) after subclavian flap-aortoplasty. Note the absence of the left subclavian artery in both images.

**Fig. (5):**
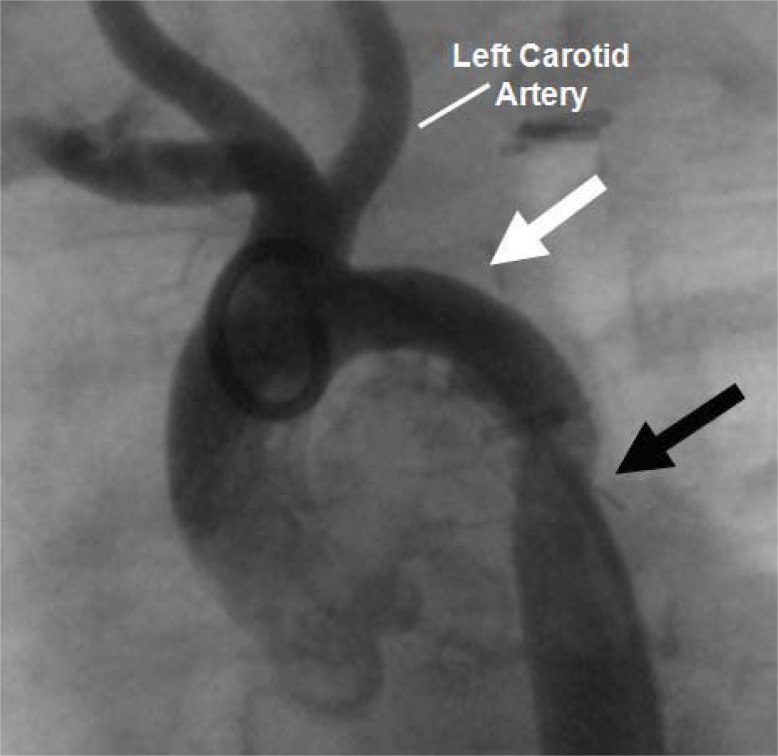
Recurrent coarctation shown in this 1-year old patient who previously underwent repair by subclavian flap aortoplasty. The white arrow demonstrates the absent left subclavian artery, while the black arrow demonstrates the recurrent coarctation. Balloon angioplasty successfully reduced the gradient across the obstruction from 80 mmHg to 15mmHg. This patient further has a common origin of the left carotid artery and the innominate artery, a common aortic arch variant.

Initial proponents of this technique touted lower recoarctation rates in infants because of the use of one’s native tissue to perform the repair [39, 40]. Medium term follow-up, however, demonstrated upwards of a 23% recurrence rate with some aneurysm formation for patients operated on during infancy [41] (Fig. **4**). Recent long-term studies have demonstrated similar rates of recoarctation from neonatal repair, but also have shown much lower recurrence rates when the operation was performed on older children (0-3%) [42, 43] (Fig. **5**). While severe left arm ischemia is rare, long-term arm length discrepancy and claudication with exercise due to compromised arterial supply has clearly been described [42, 44]. 

### Coarctation Resection with Extended End-to-End Anastomosis

The preferred method in most surgical centers, coarctation resection with extended end-to-end anastomosis was first described by Amato in 1977 [45] and is currently thought to better deal with residual ductal tissue in addition to the frequently encountered problem of transverse arch hypoplasia. The technique is largely performed through a lateral thoracotomy but occasionally requires a midline sternotomy when more complex reconstructions for aortic arch hypoplasia or other intracardiac repairs are warranted. The proximal clamp is placed across the left subclavian artery or the left carotid obliquely across the transverse arch with the distal clamp being situated inferior to the coarctation site. The ductus arteriosus is then ligated and divided. The coarctation tissue and probable remnant ductal tissue is excised with a beveled incision and the proximal arch is filleted open with extension to the undersurface of the transverse arch. A suture anastomosis is then performed after significant mobilization of the descending aorta. 

The most recent data suggests low mortality, shorter cross-clamp times, and lower recoarctation rates at 4-13% looking at patients 5-10 years post-surgery [46-52]. The procedure has low mortality even on patients under two kilograms [53] and has been thought to maintain long-term aortic compliance [54]. Variations on this technique, including end-to-side anastomosis, have demonstrated similar results [46, 49, 55]. 

### Coarctation Resection with Interposition Graft

**Fig. (6):**
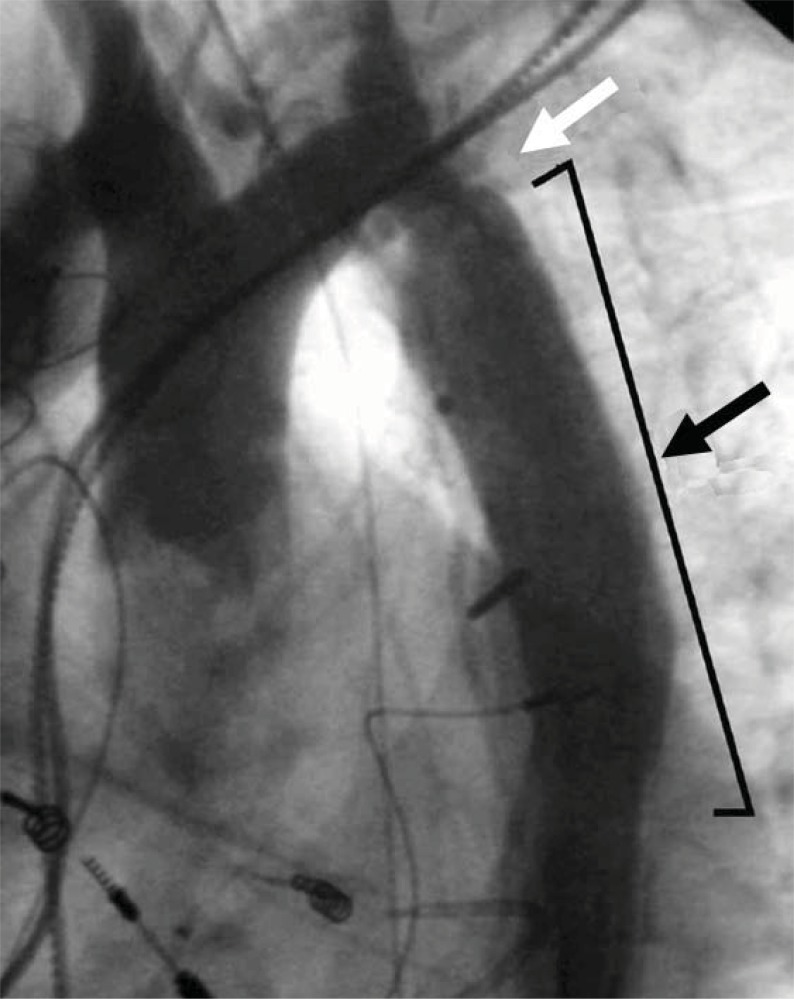
This patient with a ventricular septal defect and coarctation of the aorta previously underwent ventricular septal defect surgical patch closure and coarctation repair. Recurrence of the coarctation in a long-segment, however, necessitated placement of an interposition graft (black arrow). Recurrent obstruction is seen at the proximal end of the graft (white arrow). Note pacemaker wires related to post-surgical heart block.

This technique, while initially used by Gross [56], has been reserved for patients in whom outgrowth of the graft is not a concern, or in patients with long-segment coarctation. After the aorta is cross-clamped and the obstructive tissue resected, a tube graft of either aortic homograft [56] or Dacron [57] is sewn into the aorta, creating an unobstructed path for blood (Fig. **6**). The main disadvantage of this technique is that it requires a longer cross-clamp time for two surgical anastomoses to be sewn, and the tube graft will not grow with the patient. Yet, for adult-sized patients presenting with long-segment coarctation, this technique may be preferable at many centers.

## CURRENT AND FUTURE TRANSCATHETER THERAPIES

**Fig. (7):**
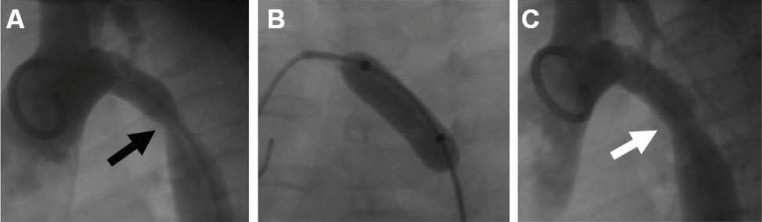
This 1 year-old infant presented with post-operative recurrent coarctation of the aorta (Panel A, black arrow) after patch aortoplasty. A high-pressure angioplasty balloon is inflated across the obstructed segment (Panel B), with improvement angiographically (Panel C) and hemodynamically (pressure gradient reduced from 55 mmHg to 10 mmHg).

Initially described by Singer in 1982 [58] and Lock in 1983 [59], transcatheter balloon angioplasty has been extensively studied as an alternative to surgical intervention. A balloon angioplasty catheter is advanced retrograde into the aorta across the stenotic area. It is inflated under high pressure to disrupt the intima and medial layers of the coarcted segment [60]. After the balloon is deflated, intraluminal blood pressure causes beneficial remodeling and expansion of the stenotic segment (Fig. **7**). 

While procedural success and safety in infants with native coarctation is quite good [61], recurrence rates as high as 80% have been described just a few months post angioplasty [62-65]. Results for native coarctation in adolescents and adults are significantly better with lower recurrence than published surgical rates in the short-term [66-68]. In the long-term, however, direct comparison of surgery and balloon angioplasty for adolescents and adults demonstrates higher recurrence rates and aneurysm formation for angioplasty but higher procedural complications for surgery [69-71]. 

These results suggest that for native discrete coarctation it is reasonable to initially pursue balloon angioplasty for older children and adults. In infants, however, transcatheter procedures should be considered only a temporizing step prior to eventual surgical repair in select high-risk patients who would benefit from more time before surgery. For recurrent coarctation, however, balloon angioplasty is generally felt to be superior to surgical reintervention due to its high procedural success compared to a greater incidence of surgical complications [72, 73].

**Fig. (8):**
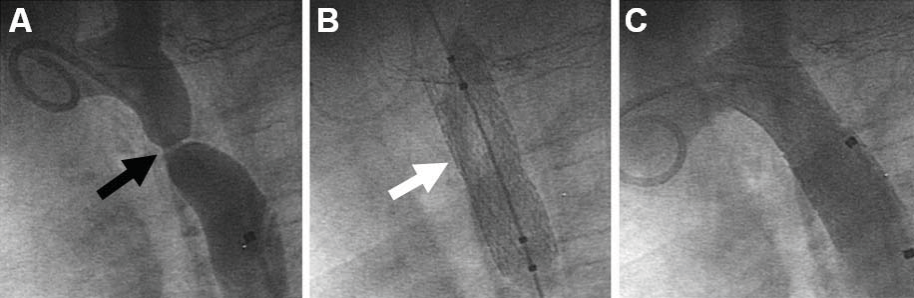
This 8 year-old patient with native coarctation of the aorta, as shown in Panel A, had a 50 mmHg gradient across the narrowed segment (black arrow). He underwent angioplasty with stent placement (Panel B, white arrow), completely eliminating the pressure gradient as well as the anatomic obstruction (Panel C).

**Fig. (9):**
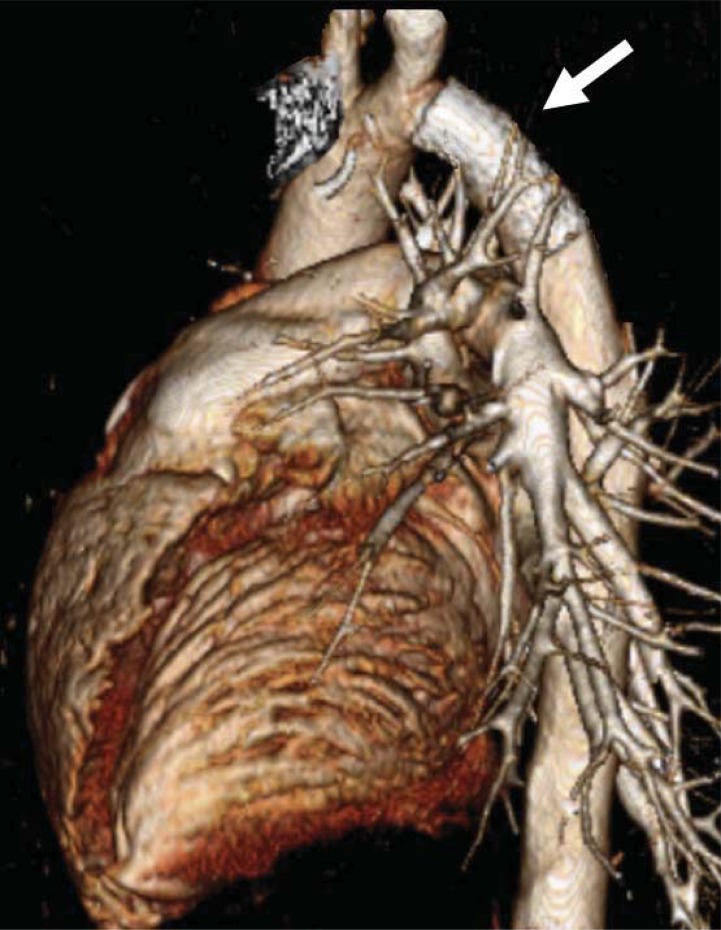
Computed tomography scan with three-dimensional reconstruction in a 22 year-old patient with recurrent coarctation. Note the presence of an endovascular stent in the descending aorta (white arrow) with no proximal aneurysm formation.

For discrete coarctations, balloon angioplasty appears to work quite well, but elastic recoil makes angioplasty for longer hypoplastic segments less successful. Interventional cardiologists have thus begun placing balloon expandable stents across thoracic and abdominal coarctations to improve procedural outcomes [74, 75] (Fig. **8**). Acute and intermediate results in older children and adults for both native and recurrent coarctation have shown this technique to be both safe and effective with similar reintervention rates to surgery and fewer complications [76-80]; often, the reintervention necessary is simply redilation of the previously-placed stent. The most frequent complication from this procedure is aneurysm formation from aortic wall injury, which can occur in up to 6% of these patients [76, 78, 79]. Magnetic resonance imaging in patients with stents produces significant artifact, making computed tomography a better modality for monitoring for recurrent obstructions (Fig. **9**). 

Stent implantation in younger children and infants is limited due to concerns for somatic growth of the younger aorta. Traditional stents have been tried with eventual redilation, but long-term outcomes of this approach are lacking [81, 82]. “Open-ring” or growth stents have been suggested as a way to implant stents in infants and young children [83, 84]. These stents can be successfully overdilated to allow for somatic growth, but long-term data on their use is not available. 

New on the horizon are biodegradable stents that are made of polymers that will absorb into the body approximately 3-6 months after implantation and are not affected by somatic growth. At the time of absorption, the hope is that enough native coarctation tissue is destroyed making reintervention risks low. A magnesium polymer stent and a Poly-L-Lactic Acid (PLLA) stent [85-87] are two types of bioabsorbable devices that have previously shown promise for coronary disease in adults. While the magnesium polymer stent did not pan out for use in coarctation, the PLLA stent appears to be quite effective but has not yet been tested in large groups of young patients. 

## LONG-TERM CONSIDERATIONS

Regardless of the specific intervention, the aorta in patients with coarctation is truly abnormal post-procedure and may be at risk for to specific complications long-term. Chronic hypertension remains present in a significant number of individuals despite improvement in anatomic obstruction [67, 79, 88, 89]. Recurrence or persistence of aortic coarctation occurs in a small, but significant, percentage of patients regardless of the type of therapeutic approach and how long ago the intervention was performed. Consequently, even patients with isolated and uncomplicated aortic coarctation require serial upper and lower extremity blood pressure measurements as part of life-long follow-up. 

Individuals with long-segment arch hypoplasia such as those with hypoplastic left heart syndrome warrant even closer monitoring for recurrent coarctation since ventricular function can deteriorate with persistent obstruction [90]. These fragile patients respond well initially to balloon angioplasty for recurrent coarctation but still have a higher rate of surgical intervention at the time of their next operation [36, 91]. 

As described earlier, aneurysm development has been noted after transcatheter balloon valvuloplasty, endovascular stenting [78, 79] and to a lesser extent after surgical approach [92]. They have been identified more commonly by magnetic resonance imaging and angiography, but even plain chest films have high sensitivity for detecting aneurysms [29]. The use of covered stents has recently been suggested as a transcatheter approach to handling aortic aneurysms in the setting of coarctation [93]. 

Endocarditis has been described in the past for patients with native coarctation [94], after surgical repair of coarctation [95] and also post balloon angioplasty [96]. Life-long endocarditis prophylaxis used to be recommended for patients with residual anatomic abnormalities who were undergoing dental work. In 2007, new standardized recommendations were made that limit prophylactic antibiotic use in patients with unrepaired or repaired coarctation. Currently, only those patients who had their coarctations repaired with prosthetic material require endocarditis prophylaxis for a period of 6 months post-procedure [97].

## CONCLUSION

There have been significant advances in transcatheter techniques for aortic coarctation in the last thirty years and future innovations are continuing to be developed. Surgical approaches, as well, have evolved to the point of extremely low mortality and infrequent morbidity on even the smallest of infants. With both approaches, there are definitive issues of recoarctation, aneurysm formation and persistent hypertension that require life-long monitoring. Still, aspects of individual patient’s anatomy will continue to drive decision-making for all therapeutic interventions. Continued assessment of long-term data surrounding the most recent transcatheter therapies and the current surgical approaches is necessary to further validate each option. 

## References

[1] Kieth J, Rowe  R, Vlad P (1958). Heart Disease in Infancy and Childhood.

[2] Anderson RH, Lenox CC, Zuberbuhler JR (1983). Morphology of ventricular septal defect associated with coarctation of aorta. Brit Heart J.

[3] Folger GM, Stein PD (1984). Bicuspid aortic valve morphology when associated with coarctation of the aorta. Catheter Cardiovasc Diag.

[4] Shone JD, Sellers RD, Anderson RC, Adams P, Lillehei CW, Edwards J (1963). The developmental complex of “parachute mitral valve,”
supravalvular ring of left atrium, subaortic stenosis, and coarctation
of aorta. Am J Cardiol.

[5] Machii M, Becker A (1995). Nature of coarctation in hypoplastic left heart syndrome. Ann Thorac Surg.

[6] Morgagni G (1760). De sedibus et causis morborum. Epist XVIII.

[7] Crafoord C, Nylin G (1945). Congenital coarctation of the aorta and its surgical treatment. J Thorac Cardiovasc Surg.

[8] Gross R, Hufnagel C (1945). Coarctation of the aorta. Experimental studies
regarding its surgical correction. N Engl J Med.

[9] Russell G, Berry P, Watterson K, Dhasmana J, Wisheart J (1991). Patterns of ductal tissue in coarctation of the aorta in the first three months of life. J Thorac Cardiovasc Surg.

[10] Rudolph A, Heymann M, Spitznas U (1972). Hemodynamic considerations in the development of narrowing of the aorta. Am J Cardiol.

[11] Campbell M (1970). Natural history of coarctation of the aorta. Br Heart J.

[12] Stern H, Locher D, Wallnofer K (1991). Noninvasive assessment of coarctation
of the aorta: comparative measurements by two-dimensional
echocardiography, magentic resonance, and angiography. Pediatr Cardiol.

[13] Shaddy R, Snider A, Silverman N, Lutin W (1986). Pulsed Doppler findings in patients with coarctation of the aorta. Circulation.

[14] Tawes R, Aberdeen E, Waterston D, Carter R (1969). Coarctation of the
aorta in infants and children. A review of 333 operative cases, including
179 infants. Circulation.

[15] Fawzy M, von Sinner W, Rifai A (1993). Magnetic resonance imaging
compared with angiography in the evaluation of intermediateterm
result of coarctation balloon angioplasty. Am J Cardiol.

[16] Mohiaddin R, Kilner P, Rees S, Longmore D (1993). Magnetic resonance
volume flow and jet velocity mapping in aortic coarctation. J Am Coll Cardiol.

[17] Oshinski J, Parks W, Markou C (1996). Improved Measurement of Pressure Gradients in Aortic Coarctation by Magnetic Resonance Imaging. J Am Coll Cardiol.

[18] Rees S, Somerville J, Ward C (1989). Coarctation of the aorta: MR imaging in late postoperative assessment. Radiology.

[19] Shih M-CP, Tholpady A, Kramer CM, Sydnor MK, Hagspiel KD (2006). Surgical and endovascular repair of aortic coarctation: normal findings and appearance of complications on CT angiography and MR angiography. Am J Roent.

[20] Nielsen JC, Powell AJ, Gauvreau K, Marcus EN, Prakash A, Geva T (2005). Magnetic resonance imaging predictors of coarctation severity. Circulation.

[21] Araoz P, Reddy G, Tarnoff H, Roge C, Higgins C (2003). MR findings of collateral circulation are more accurate measures of hemodynamic significance than arm-leg blood pressure gradient after repair of coarctation of the aorta. J Magn Reson Imaging.

[22] Williams W, Shindo G, Trusler G, Dische M, Olley P (1980). Results of repair of coarctation of the aorta during infancy. J Thorac Cardiovasc Surg.

[23] Kappetein A, Zwinderman A, Bogers A, Rohmer J, Huysmans H (1994). More than thirty-five years of coarctation repair. An unexpected
high relapse rate. J Thorac Cardiovasc Surg.

[24] Hesslein P, McNamara D, Morriss M, Hallman G, Cooley D (1981). Comparison of resection versus partch aortoplasty for repair of coarctation in infants and children. Circulation.

[25] Ziemer G, Jonas R, Perry S, Freed M, Castaneda A (1986). Surgery for coarctation of the aorta in the neonate. Circulation.

[26] Vossschulte K (1961). Surgical correction of coarctation of the aorta by an “isthmusplastic” operation. Thorax.

[27] Maxey T, Serfontein S, Reece T, Rheuban K, Kron I (2003). Transverse arch hypoplasia may predispose patients to aneurysm formation after patch repair of aortic coarctation. Ann Thorac Surg.

[28] Venturini A, Perna A, Bianchi G (1978). Repair of coarctation of the thoracic
aorta without resection. Patch graft aortoplasty. Follow-up
study of 46 cases. J Cardiovasc Surg.

[29] Bromberg B, Beekman R, Rocchini A (1989). Aortic aneurysm after patch
aortoplasty repair of coarctation: a prospective analysis of prevalence,
screening tests and risks. J Am Coll Cardiol.

[30] Rheuban K, Carpenter M, Jedeikin R (1985). Aortic aneurysm after patch
aortoplasty for coarctation in childhood. Am J Cardiol.

[31] Rheuban K, Gutgesell H, Carpenter M (1986). Aortic aneurysm after patch angioplasty for aortic isthmic coarctation in childhood. Am J Cardiol.

[32] Backer C, Paape K, Zales V, Weigel T, Mavroudis C (1995). Coarctation
of the aorta. Repair with polytetrafluoroethylene patch aortoplasty. Circulation.

[33] Walhout R, Lekkerkerker J, Oron G, Hitchcock F, Meijboom E, Bennink G (2003). Comparison of polytetrafluoroethylene patch aortoplasty and end-to-end anastomosis for coarctation of the aorta. J Thorac Cardiovasc Surg.

[34] Bove E, Ohye R, Devaney E (2004). Hypoplastic left heart syndrome:
conventional surgical management. Semin Thorac Cardiovasc Surg
Pediatr Card Surg Annu.

[35] Ashcraft T, Jones K, Border W (2008). Factors affecting long-term risk of aortic arch recoarctation after the Norwood procedure. Ann Thorac Surg.

[36] Porras D, Brown D, Marshall A, del Nido P, Bacha E, McElhinney D (2011). Factors associated with subsequent arch reintervention after initial balloon aortoplasty in patients with Norwood procedure and arch obstruction. J Am Coll Cardiol.

[37] Waldhausen J, Nahrwold D (1966). Repair of coarctation of the aorta with a subclavian flap. J Thorac Cardiovasc Surg.

[38] Kanter K, Vincent R, Fyfe D (2001). Reverse subclavian flap repair of hypoplastic transverse aorta in infancy. Ann Thorac Surg.

[39] Midgley F, Scott L, Perry L, Shapiro S, McClenathan J (1978). Subclavian flap aortoplasty for treatment of coarctation in early infancy. J Pediatr Surg.

[40] Sousa A, Bos E, Elzenga N, de Villeneuve V (1983). Subclavian flap aortoplasty for treatment of coarctation of the aorta in infants less than 3 months of age. Thorac Cardiovasc Surg.

[41] Beekman R, Rocchini A, Behrendt D (1986). Long-term outcome after repair of coarctation in infancy: Subclavian angioplasty does not reduce the need for reoperation. J Am Coll Cardiol.

[42] Pandey R, Jackson M, Ajab S, Gladman G, Pozzi M (2006). Subclavian flap repair: review of 399 patients at median follow-up of fourteen years. Ann Thorac Surg.

[43] Barreiro CJ, Ellison TA, Williams JA, Durr ML, Cameron DE, Vricella LA (2007). Subclavian flap aortoplasty: still a safe, reproducible,
and effective treatment for infant coarctation. Eur J Cardiothorac Surg.

[44] van Son J, van Asten W, van Lier H (1990). Detrimental sequelae on the hemodynamics of the upper left limb after subclavian flap angioplasty in infancy. Circulation.

[45] Amato J, Rheinlander H, Cleveland R (1977). A method of enlarging the distal transverse arch in infants with hypoplasia and coarctation of the aorta. Ann Thorac Surg.

[46] Thomson JDR, Mulpur A, Guerrero R, Nagy Z, Gibbs JL, Watterson KG (2006). Outcome after extended arch repair for aortic coarctation. Heart.

[47] Hager A, Schreiber C, Nutzl S, Hess J (2009). Mortality and restenosis rate of surgical coarctation repair in infancy: a study of 191 patients. Cardiology.

[48] Burch PT, Cowley CG, Holubkov R (2009). Coarctation repair in neonates and young infants: is small size or low weight still a risk factor?. J Thorac Cardiovasc Surg.

[49] Tabbutt S, Nicolson SC, Dominguez TE (2008). Perioperative course in 118 infants and children undergoing coarctation repair via a thoracotomy: a prospective, multicenter experience. J Thorac Cardiovasc Surg.

[50] Wright GE, Nowak CA, Goldberg CS, Ohye RG, Bove EL, Rocchini AP (2005). Extended resection and end-to-end anastomosis for aortic coarctation in infants: results of a tailored surgical approach. Ann Thorac Surg.

[51] Kumar TKS, Zurakowski D, Sharma R, Saini S, Jonas R (2011). Prediction of recurrent coarctation by early postoperative blood pressure gradient. J Thorac Cardiovasc Surg.

[52] Brown JW, Ruzmetov M, Hoyer MH, Rodefeld MD, Turrentine MW (2009). Recurrent coarctation: is surgical repair of recurrent coarctation of the aorta safe and effective?. Ann Thorac Surg.

[53] Sudarshan CD, Cochrane AD, Jun ZH, Soto R, Brizard CP (2006). Repair of coarctation of the aorta in infants weighing less than 2 kilograms. Ann Thorac Surg.

[54] Bassareo PP, Marras AR, Manai ME, Mercuro G (2009). The influence of different surgical approaches on arterial rigidity in children after aortic coarctation repair. Pediatr Cardiol.

[55] Younoszai AK, Reddy VM, Hanley FL, Brook MM (2002). Intermediate term follow-up of the end-to-side aortic anastomosis for coarctation of the aorta. Ann Thorac Surg.

[56] Gross R (1951). Treatment of certain aortic coarctations by homologous grafts; a report of nineteen cases. Ann Surg.

[57] Aris A, Subirana M, Ferres P, Torner-Soler M (1999). Repair of arotic coarctation in patients more than 50 years of age. Ann Thorac Surg.

[58] Singer M, Rowen M, Dorsey T (1982). Transluminal aortic balloon angioplasty for coarctation of the aorta in the newborn. Am Heart J.

[59] Lock J, Bass J, Amplatz K, Fuhrman B, Castaneda-Zuniga W (1983). Balloon dilation angioplasty of aortic coarctations in infants and children. Circulation.

[60] Lock J, Niemi T, Burke B, Einzig S, Castaneda-Zuniga W (1982). Transcutaneous angioplasty of experimental aortic coarctation. Circulation.

[61] Tynan M, Finley J, Fontes V, Hess J, Kan J (1990). Balloon angioplasty for the treatment of native coarctation: Results of Valvuloplasty and Angioplasty of Congenital Anomalies Registry. Am J Cardiol.

[62] Rothman A, Galindo A, Evans WN, Collazos JC, Restrepo H (2010). Effectiveness and safety of balloon dilation of native aortic coarctation in premature neonates weighing < or = 2,500 grams. Am J Cardiol.

[63] Suarez de Lezo J, Pan M, Romero M (2005). Percutaneous interventions on severe coarctation of the aorta: a 21-year experience. Pediatr Cardiol.

[64] Rao S, Jureidini S, Balfour I, Singh G, Chen S (2003). Severe aortic coarctation in infants less than 3 months: successful palliation by balloon angioplasty. J Invasive Cardiol.

[65] Fiore AC, Fischer LK, Schwartz T (2005). Comparison of angioplasty and surgery for neonatal aortic coarctation. Ann Thorac Surg.

[66] Reich O, Tax P, Bartáková H (2008). Long-term (up to 20 years) results of percutaneous balloon angioplasty of recurrent aortic coarctation without use of stents. Eur Heart J.

[67] Fawzy ME, Awad M, Hassan W, Al Kadhi Y, Shoukri M, Fadley F (2004). Long-term outcome (up to 15 years) of balloon angioplasty of discrete native coarctation of the aorta in adolescents and adults. J Am Coll Cardiol.

[68] Hassan W, Awad M, Fawzy ME (2007). Long-term effects of balloon
angioplasty on left ventricular hypertrophy in adolescent and
adult patients with native coarctation of the aorta. Up to 18 years
follow-up results. Catheter Cardiovasc Interv.

[69] Cowley CG, Orsmond GS, Feola P, McQuillan L, Shaddy RE (2005). Long-term, randomized comparison of balloon angioplasty and
surgery for native coarctation of the aorta in childhood. Circulation.

[70] Rodés-Cabau J, Miró J, Dancea A (2007). Comparison of surgical
and transcatheter treatment for native coarctation of the aorta in patients
> or = 1 year old. The Quebec Native Coarctation of the
Aorta study. Am Heart J.

[71] Wong D, Benson LN, Van Arsdell GS, Karamlou T, McCrindle BW (2008). Balloon angioplasty is preferred to surgery for aortic coarctation. Cardiol Young.

[72] Rao P, Galal O, Smith P, Wilson A (1996). Immediate and follow-up results of balloon angioplasty of native aortic coarctation in infants and children. J Am Coll Cardiol.

[73] Brown ML, Burkhart HM, Connolly HM, Dearani J a, Hagler DJ, Schaff HV (2010). Late outcomes of reintervention on the descending aorta after repair of aortic coarctation. Circulation.

[74] Beekman R, Muller D, Reynolds P, Moorehead C, Heidelberger K, Lupinetti F (1993). Balloon-expandable stent treatment of experimental coarctation of the aorta: early hemodynamic and pathological evaluation. J Interv Cardiol.

[75] Rosenthal E, Qureshi S, Tynan M (1995). Stent implantation for aortic recoarctation. Am Heart J.

[76] Qureshi AM, McElhinney DB, Lock JE, Landzberg MJ, Lang P, Marshall AC (2007). Acute and intermediate outcomes, and evaluation of
injury to the aortic wall, as based on 15 years experience of implanting
stents to treat aortic coarctation. Cardiol Young.

[77] Forbes TJ, Garekar S, Amin Z (2007). Procedural results and acute complications in stenting native and recurrent coarctation of the aorta in patients over 4 years of age: a multi-institutional study. Catheter Cardiovasc Interv.

[78] Forbes TJ, Moore P, Pedra C (2007). Intermediate follow-up following intravascular stenting for treatment of coarctation of the aorta. Catheter Cardiovasc Interv.

[79] Holzer R, Qureshi S, Ghasemi A (2010). Stenting of aortic coarctation:
acute, intermediate, and long-term results of a prospective
multi-institutional registry--Congenital Cardiovascular Interventional
Study Consortium (CCISC). Catheter Cardiovasc Interv.

[80] Forbes TJ, Kim DW, Du W (2011). Comparison of surgical, stent,
and balloon angioplasty treatment of native coarctation of the aorta:
an observational study by the CCISC (Congenital Cardiovascular
Interventional Study Consortium). J Am Coll Cardiol.

[81] Mohan UR, Danon S, Levi D, Connolly D, Moore JW (2009). Stent implantation for coarctation of the aorta in children <30 kg. JACC Cardiovasc Interv.

[82] Bentham J, Shettihalli N, Orchard E, Westaby S, Wilson N (2010). Endovascular
stent placement is an acceptable alternative to reoperation
in selected infants with residual or recurrent aortic arch obstruction. Catheter Cardiovasc Interv.

[83] Ing F, Fagan T, Kearney D, Al E (1996). A new “open-ring” stent (abstr). Circulation.

[84] Ewert P, Peters B, Nagdyman N, Miera O, Kuhne T, Berger F (2008). Early and Mid-Term Results With the Growth Stent—A Possible Concept for Transcatheter Treatment of Aortic Coarctation From Infancy to Adulthood by Stent Implantation?. Catheter Cardiovasc Interv.

[85] Schranz D, Zartner P, Michel-Behnke I, Akinturk H (2006). Bioabsorbable metal stents for percutaneous treatment of critical recoarctation of the aorta in a newborn. Catheter Cardiovasc Interv.

[86] Tamai H, Igaki K, Kyo E, Kosuga K (2000). Initial and 6-Month Results of Biodegradable Poly-l-Lactic Acid Coronary Stents in Humans. Circulation.

[87] Ormiston J, Serruys P (2009). Bioabsorbable coronary stents. Circ Cardiovasc Interv.

[88] Clarkson P, Nicholson M, Barratt-Boyes B, Neutze J, Whitlock R (1983). Results after repair of coarctation of the aorta beyond infancy: a 10 to 28 year follow-up with particular reference to late systemic hypertension. Am J Cardiol.

[89] Gillett C, Wong A, Wilson DG, Wolf AR, Martin RP, Kenny D (2011). Underrecognition of elevated blood pressure readings in children after early repair of coarctation of the aorta. Pediatr Cardiol.

[90] Larrazabal LA, Selamet Tierney ES, Brown DW (2008). Ventricular function deteriorates with recurrent coarctation in hypoplastic left heart syndrome. Ann Thorac Surg.

[91] Zeltser I, Menteer J, Gaynor JW (2005). Impact of re-coarctation following the Norwood operation on survival in the balloon angioplasty era. J Am Coll Cardiol.

[92] Shaddy R, Boucek M, Sturtevant J (1993). Comparison of angioplasty and surgery for unoperated coarctation of the aorta. Circulation.

[93] Tzifa A, Ewert P, Brzezinska-Rajszys G (2006). Covered Cheatham-platinum stents for aortic coarctation: early and intermediate-term results. J Am Coll Cardiol.

[94] Franco-Paredes C, Workowski K, Harris M (2002). Infective endocarditis-endarteritis complicating coarctation of the aorta. Am J Med.

[95] Villagra F, Rufilanchas J, Tellez G, Maronas J, Iglesias A, Aymerich D (1977). Early and late death of surgically treated patients with coarctation of the aorta. Vasc Surg.

[96] Sanyal S, Wilson N, Twum-Danso K, Abomelha A, Sohel S (1990). Moraxella endocarditis following balloon angioplasty of aortic coarctation. Am Heart J.

[97] Wilson W, Taubert K a, Gewitz M (2007). Prevention of infective endocarditis: guidelines from the American Heart Association. Circulation.

